# Immature ***Citrus unshiu*** fruit extracts inhibit adipogenesis in 3T3-L1 adipocytes via AMPK and MAPK signaling pathways

**DOI:** 10.1371/journal.pone.0322619

**Published:** 2025-05-08

**Authors:** Min Gun Kim, Kyung-Hwan Boo, Jae-Hoon Kim, Chang Sook Kim

**Affiliations:** 1 Faculty of Biotechnology, Jeju National University, Jeju, Republic of Korea; 2 Subtropical/tropical Organism Gene Bank, Jeju National University, Jeju, Republic of Korea; Cairo University, EGYPT

## Abstract

In Korea, immature citrus fruits have been extensively explored for their potential utility as functional bio-health materials owing to their various bioactive properties. However, the specific mechanisms by which they exert inhibitory effects on adipogenesis remain unclear. Therefore, this study aimed to examine the anti-obesity effects of 70% ethanol extracts of immature *Citrus unshiu* fruits and their solvent fractions (n-hexane, ethyl acetate, n-butanol, and water) on 3T3-L1 cells, as well as to explore the underlying molecular mechanisms. Additionally, this study was conducted to identify the bioactive components responsible for the anti-obesity effects. Among the fractions, the hexane fraction exhibited the most potent inhibitory effect on lipid accumulation in 3T3-L1 cells without inducing cytotoxicity. Notably, this effect was concentration-dependent. This fraction also inhibited adipogenesis during the differentiation of 3T3-L1 preadipocytes by downregulating the expression of CCAAT/enhancer-binding proteins (C/EBP), peroxisome proliferator-activated receptor-γ (PPARγ), sterol regulatory element-binding protein (SREBP), fatty acid synthase (FAS), and fatty acid binding protein 4 (FABP4). Moreover, the hexane fraction modulated the activity of AMP-activated protein kinase (AMPK) and mitogen-activated protein kinase (MAPK), both of which play critical roles in lipid metabolism. Specifically, it induced AMPK activation while downregulating MAPK signaling. Phytochemical analysis identified phytol, hexatriacontane, tangeretin, and nobiletin as the main bioactive components responsible for the observed anti-obesity effects of ICE. Overall, our results revealed that ICE exhibited notable anti-obesity activity by targeting the AMPK and MAPK signaling pathways, highlighting its potential as a natural therapeutic agent for obesity management.

## Introduction

Obesity is a disorder characterized by the excessive accumulation of fat tissue in the body and is closely associated with conditions such as nonalcoholic fatty liver, cancer, cardiovascular disease, hypertension, and type 2 diabetes, serving as an indicator of metabolic syndrome [1–3]. The primary causes of obesity include excessive fat cell growth and differentiation, increased body fat accumulation owing to an imbalance between energy intake and consumption, and genetic factors [2]. Specifically, obesity results from the accumulation of triglycerides in adipose tissue and the expansion of adipose tissue, which can occur through hypertrophy (where the size of adipocytes increases) or hyperplasia (where the number of adipocytes increases from preadipocytes).

Effective anti-obesity formulations should aim to reduce weight without adverse effects; however, most current anti-obesity drugs are associated with significant side effects, including constipation, thirst, headaches, and insomnia [4,5]. Therefore, in recent years, there has been a growing interest in developing functional health foods and pharmaceuticals that incorporate anti-obesity materials derived from natural products with minimal side effects [6,7]. Plant extracts containing polyphenols, resveratrol, and curcumin are recognized for their anti-adipogenic properties [8,9]. For example, extracts of black garlic, green tea, turmeric, and *Ecklonia cava* have been reported to inhibit acetyl-CoA carboxylase (ACC) activity by activating AMP-activated protein kinase (AMPK) and suppress fat accumulation in 3T3-L1 cells by regulating the expression of peroxisome proliferator-activated receptor-γ (PPARγ) and CCAAT/enhancer-binding protein α (C/EBPα) [10–13].

Citrus fruits, including Citrus unshiu, contain a substantial amount of physiologically active substances, such as phenolic compounds and vitamins, and thus exhibit various physiological activities, including anticancer, anti-inflammatory, and antioxidant effects [14,15]. Studies have shown that immature citrus fruits contain higher levels of flavonoids, such as narirutin and hesperidin, than their mature counterparts, which enhances their functional properties [16,17]. In particular, recent studies have highlighted the health benefits of immature citrus fruits, demonstrating that *Poncirus trifoliata* exhibits efficacy against colorectal cancer, while *Citrus unshiu* Markovich possesses antioxidant and anti-aging properties [18,19]. Accordingly, immature citrus fruits have been extensively explored for their potential utility as functional bio-health materials. In light of these findings, a series of studies are underway to explore the use of immature *C. unshiu* fruits as materials for the development of complex health functional foods and functional cosmetics [20]. This study was conducted to investigate the bioactive components and anti-obesity effects of ethanol extracts of immature *C. unshiu* fruits (ICE) and their solvent fractions on 3T3-L1 preadipocytes. Furthermore, this study aimed to assess the underlying mechanism of action of ICE and evaluate its inhibitory activity on lipid accumulation.

## Materials and methods

### Materials, chemicals, and reagents

All chemicals and reagents were obtained commercially. Dulbecco’s modified Eagle medium (DMEM), fetal bovine serum (FBS), bovine calf serum (BCS), and antibiotics (penicillin and streptomycin) were purchased from Gibco (Grand Island, NY, USA). Nonidet P-40 (NP-40), Oil Red O (ORO), 3-isobytyl-1-methylxanthine (IBMX), dexamethasone (DEX), and insulin were purchased from Sigma-Aldrich (St. Louis, MO, USA). Antibodies against sterol regulatory element-binding protein 1 (SREBP1) and β-actin were purchased from Santa Cruz Biotechnology (Dallas, TX, USA). Antibodies against fatty acid-binding protein 4 (FABP4), C/EBPα, PPARγ, fatty acid synthase (FAS), AMPK, phosphorylated AMPK (phospho-AMPK), ACC, phospho-ACC, p38 MAPK, phospho-p38 MAPK, extracellular signal-regulated kinase 1/2 (ERK1/2), and phospho-ERK1/2 were purchased from Cell Signaling Technology (Danvers, MA, USA).

### Preparation of citrus extracts

Dried, immature *C. unshiu* fruits were purchased from Samwon Nature Co., Ltd. (Jeju, Korea). ICE and their solvent fractions were prepared as described previously [20]. Briefly, 3.5 g of dried powder was mixed with 35 mL of 70% ethanol and subjected to ultrasonication for extraction. The ethanol extract was filtered, concentrated, and freeze-dried. Subsequently, 0.8 g of the dried extract powder was dissolved in distilled water (110 mL) and successively fractionated into three solvents (n-hexane, ethyl acetate [EtOAc], and n-butanol [n-BuOH]) using a separatory funnel to obtain the experimental solvent fractions and residues. All extracts were dissolved in methanol for use in the subsequent experiments.

### Cell culture and cell viability assay

The 3T3-L1 cell line, derived from mice, was obtained from the American Type Culture Collection (Virginia, USA). Cells were maintained in DMEM supplemented with 100 U/mL penicillin, 100 µg/mL streptomycin, and 10% BCS in an incubator set to 5% CO_2_ and 37 °C, with subculturing performed every 3–4 days.

To assess cell viability following treatment with ICE and its solvent fractions, the water-soluble tetrazolium-1 (WST-1) reagent [2-(4-iodophenyl)−3-(4-nitrophenyl)−5-(2,4-disulfophenyl)−2H-tetrazolium] (Boehringer Mannheim, Mannheim, Germany) was used, as described previously [20]. Briefly, 3T3-L1 cells were seeded into 24-well plates at a density of 3.5 × 10^4^ cells/well. After 24 h, the cells were treated with ICE and its solvent fractions in DMEM at various concentrations for 72 h under an atmosphere of 5% CO_2_ at 37 °C. Cell viability was determined by measuring absorbance at 450 nm using a microplate reader (Thermo Fisher Scientific, Finland) after WST-1 treatment. The results are expressed as a percentage relative to that of the control group.

### Cell differentiation

Differentiation of preadipocytes was performed as previously described [21], with modifications. Briefly, 3T3-L1 cells were seeded into 24-well plates at a density of 3.5 × 10^4^ cells/well in DMEM containing 10% BCS, with the culture medium replaced at intervals of 2 days. Differentiation was initiated for 2 days by replacing the culture medium with a differentiation medium containing 1 µ M DEX, 0.5 mM IBMX, 10 µg/mL insulin, and DMEM+10% FBS when the cells were in the post-confluence state (day 0). Cells were maintained for 2 days in a medium containing 1 µg/mL insulin in DMEM+10% FBS. Next, differentiation was induced for a total of 8 days by replacing the culture medium with DMEM+10% FBS every 2 days. To assess the effect of immature citrus extract on adipogenesis, ICE and its solvent fractions were added to the culture medium during the differentiation induction period.

### Oil Red O Staining

Differentiated mature 3T3-L1 adipocytes were washed twice with phosphate-buffered saline (PBS), fixed in 4% formalin solution for 1 h, and subsequently washed twice with distilled water. Next, the cells were stained with ORO solution (0.6%) for 1 h and washed with distilled water. Lipid droplet images of the stained adipocytes were captured using a Nikon microscope (ECLIPSE Ts2, Japan). To quantify the stained lipid droplets, they were dissolved in isopropanol containing 4% NP-40, and absorbance was measured at 520 nm using a microplate reader.

### Measurement of free glycerol content

Glycerol content was measured as described previously [21], with modifications. Fully differentiated 3T3-L1 cells were incubated in DMEM+2% BSA for 12 h. Subsequently, the IBMX-treated sample (positive control) and hexane fraction at concentrations of 75, 100, and 125 µg/mL were added to the DMEM+2% BSA and incubated for an additional 48 h. Glycerol content was measured using a free glycerol assay kit (Biomax, Korea) according to the manufacturer’s protocol.

### Western blot analysis

Cells were rinsed twice with PBS and subsequently lysed for 1 h using a lysis buffer comprising radioimmunoprecipitation assay buffer, a protease inhibitor cocktail (Hoffmann-La Roche Ltd., Switzerland), 2 mM sodium vanadate, 30 mM sodium pyrophosphate, and 100 mM sodium fluoride. After lysis, the samples were centrifuged at 1,300 × g for 15 min at 4 °C. Protein quantification was performed using the Bio-Rad protein assay kit (Bio-Rad Laboratories, USA). Equal amounts of protein were separated on a 12% sodium dodecyl sulfate-polyacrylamide gel electrophoresis gel and transferred on to polyvinylidene fluoride membranes (Millipore Corp., USA). To prevent non-specific binding, the membranes were blocked by incubating them in a solution of 5% skim milk in Tris-buffered saline with 0.05% Tween-20 (TBS-T) for 12 h at 4 °C. After blocking, the membranes were incubated with primary antibodies diluted at a ratio of 1:1000 in TBS-T for 1 h, followed by three washes with TBS-T. Subsequently, the membranes were incubated with secondary antibodies for 1 h at room temperature. Immunodetection was performed using the ECL western blotting detection reagent, and the results were visualized using the ChemiDoc MP Imaging system (Bio-Rad Laboratories, USA).

### Gas chromatography-mass spectrometry analysis

Following the protocol from our previous study [20], quantitative chromatographic analysis of the hexane fraction was conducted using gas chromatography-mass spectrometry (GC-MS) QP 2010 Plus (Shimadzu Corp., Kyoto, Japan) equipped with an Agilent high-performance MS 5 column (30 m × 0.25 mm × 0.5 µm). Samples were injected in splitless mode at 230 °C, with helium serving as the carrier gas at a constant flow rate of 1 mL/min. The operating temperatures were set as follows: the detector at 250 °C and the column oven ramping from 100 °C to 300 °C at a rate of 10 °C/min. Mass spectral data were analyzed using the GC-MS library database (Wiley, version 9).

### Statistical analysis

Data are presented as the mean ± standard deviation of at least three experiments. One-way analysis of variance was used to compare the data obtained from individual groups. Significant values are indicated by asterisks (*p < 0.05, **p < 0.01, and ***p < 0.001).

## Results

### Cytotoxic effect of ICE and its solvent fractions on 3T3-L1 adipocytes

**Fig 1:**
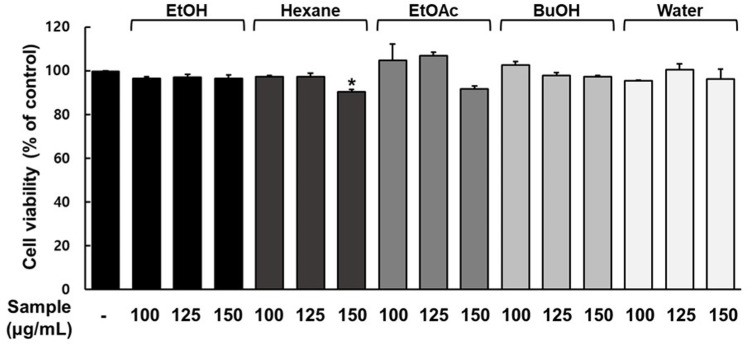
Inhibitory effect of immature Citrus unshiu fruit extract (ICE) and its solvent fractions (n-Hexane, EtOAc, BuOH, H_2_O) on cell viability of 3T3-L1 preadipocytes. The values are expressed as mean±SD (n = 3), * p < 0.05 compared with non-treated cells.

The effects of ICE and its solvent fractions (n-hexane, EtOAc, n-BuOH, and water) on cell viability in 3T3-L1 preadipocytes were measured using the WST-1 assay. Treatment of 3T3-L1 adipocytes with ICE at concentrations up to 500 µg/mL did not result in notable cytotoxic effects compared with that in the untreated control group (Fig 1). However, the WST-1 assay indicated that the n-hexane fraction at 150 µg/mL induced some level of cell toxicity. Accordingly, the highest investigational concentrations for the ICE and solvent fraction samples were established at 500 µg/mL and 125 µg/mL, respectively.

### Effect of ICE and its solvent fractions on preadipocyte differentiation and lipid accumulation

**Fig 2:**
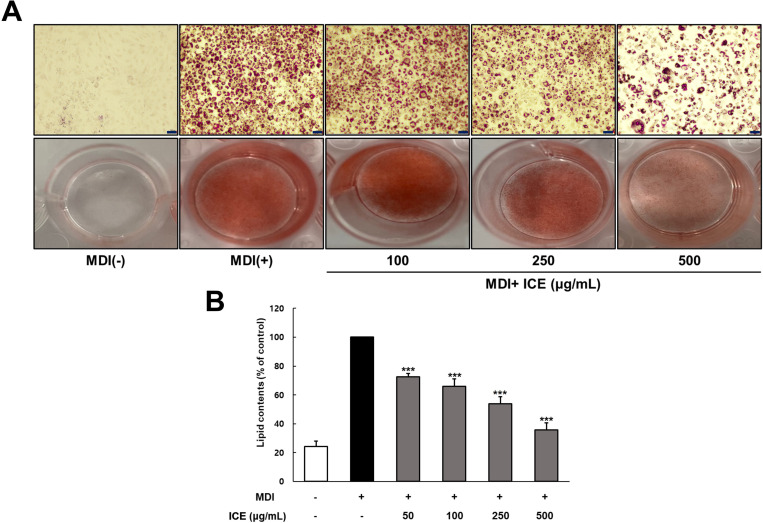
Effect of ICE on differentiation of 3T3-L1 preadipocytes. Two-day post confluent 3T3-L1 cells differentiated with MDI medium (1 μM DEX, 0.5 mM IBMX, 10 µ g/mL insulin) in the present or absence ICE. **(A)** Differentiated adipocytes were stained with Oil Red O on day 8 and stained triglycerides were presented at x200 magnification (scale bar: 100 μm). **(B)** Lipid contents were measured at 520 nm by microplate reader. The values represents means±SDs of triplicate experiments. Significant values are represented by asterisks (*** p < 0.001). Signification of the values is calculated in comparison to the values obtained using MDI-treated cells.

**Fig 3:**
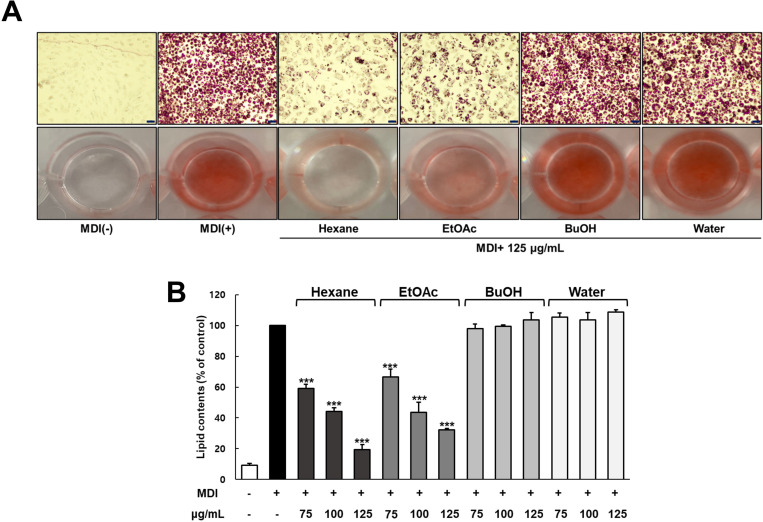
Effect of ICE fractions (hexane, EtOAc, BuOH, H_2_O) on differentiation of 3T3-L1 preadipocytes. Two-day post confluent 3T3-L1 cells differentiated with MDI medium (1 μM DEX, 0.5 mM IBMX, 10 µ g/mL insulin) in the present or absence ICE. **(A)** Differentiated adipocytes were stained with Oil Red O on day 8 and stained triglycerides were presented at x200 magnification (scale bar: 100 μm). **(B)** Lipid contents were measured at 520 nm by microplate reader. The values represents means±SDs of triplicate experiments. Significant values are represented by asterisks (*** p < 0.001). Signification of the values is calculated in comparison to the values obtained using MDI-treated cells.

To evaluate the effect of ICE on preadipocyte differentiation and lipid accumulation, cells were treated with the experimental samples and allowed to differentiate for 8 days, followed by ORO staining to assess the extent of lipid accumulation (Fig 2A). The control group exhibited cell differentiation and lipid droplet formation induced by MDI treatment compared with the normal group (Fig 2). However, ICE treatment significantly inhibited preadipocyte differentiation and lipid droplet formation compared with that in the control group. The suppressive effects of ICE on lipid accumulation were dose-dependent, with inhibitory effects at concentrations of 50, 100, 250, and 500 µg/mL estimated at 27%, 34%, 46%, and 64%, respectively, relative to those of the control (p < 0.001) (Fig 2B). The effects of the solvent fractions of ICE on preadipocyte differentiation and lipid accumulation were also assessed. At a concentration of 125 µg/mL, both the n-hexane and EtOAc fractions markedly reduced preadipocyte differentiation and lipid droplet formation compared with the control treatment (Fig 3A). Notably, the hexane fraction demonstrated greater inhibitory effects on differentiation and lipid droplet accumulation than the other solvent fractions. Furthermore, intracellular triglyceride (TG) content was measured using ORO quantification (Fig 3B and S1 Fig). Excluding the BuOH and Water fractions, the n-hexane and EtOAc fractions effectively inhibited differentiation. In particular, both the n-hexane and EtOAc fractions demonstrated strong, dose-dependent inhibitory effects, with the n-hexane fraction reducing differentiation by approximately 80% at a non-toxic concentration of 125 µg/mL.

### Effects of ICE hexane fraction on the expression of adipogenic key transcription factors and adipogenesis-related proteins

**Fig 4:**
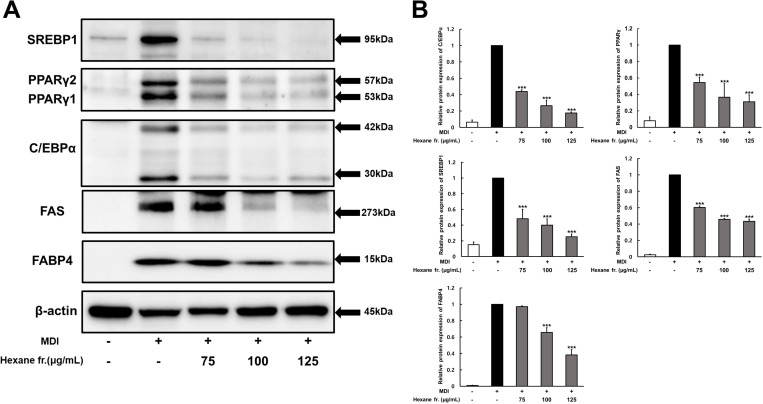
Effect of hexane fraction of immature Citrus unshiu fruits on the expression of SREBP, PPAR **γ,** **C/EBPα,** **FAS and FABP4 during 3T3-L1 preadipocyte differentiation.** Two-day post confluent 3T3-L1 cells differentiated with MDI plus various concentration hexane fractions, respectively. **(A)** Western blot analysis measured the SREBP, PPARγ, C/EBPα, FAS, FABP4 and β-actin expression. **(B)** The intensity of each band was determined using image **J.** The values represents means±SDs of triplicate experiments. Significant values are represented by asterisks (*** p < 0.001). Signification of the values is calculated in comparison to the values obtained using MDI-treated cells.

Adipocyte differentiation is regulated by complex signaling cascades involving transcription factors such as PPARγ and C/EBPα. Therefore, western blotting analysis was conducted to explore whether the hexane fraction affected the protein expression of key adipogenic transcription factors, including SREBP1, PPARγ, and C/EBPβ. Additionally, the expression levels of their target genes, FAS and FABP4, were assessed. MDI-stimulated control cells exhibited a marked increase in crucial adipogenic transcription factors such as SREBP1, PPARγ, and C/EBPα, as well as their target genes FAS and FABP4 (Fig 4A). However, the experimental group treated with the hexane fraction exhibited a concentration-dependent decrease in the expression of SREBP1, PPARγ, and C/EBPα (Fig 4B). Additionally, treatment with the hexane fraction at concentrations of 75–125 µg/mL effectively reduced the expression of proteins involved in adipogenesis. Specifically, the hexane fraction treatment significantly reduced the protein expression levels of FAS and FABP4 by 54.3% (p < 0.001) and 34.4% (p < 0.001), respectively, at 100 µg/mL, and by 57.8% and 61.7%, respectively, at 125 µg/mL.

### Effects of ICE hexane fraction on the regulation of AMPK and MAPK signaling

**Fig 5:**
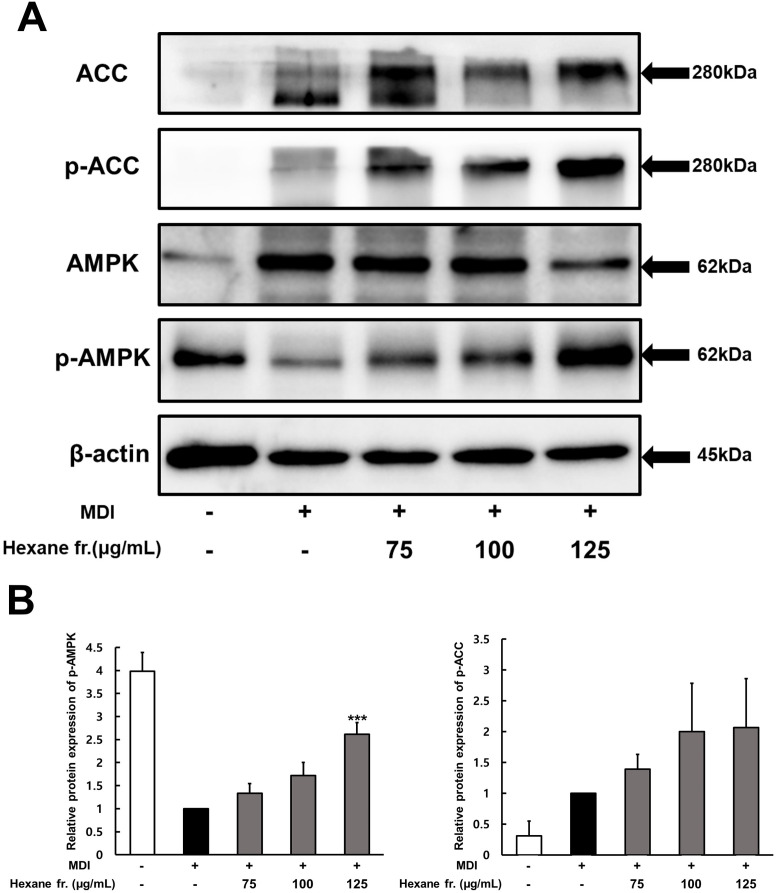
Effect of hexane fraction of immature Citrus unshiu fruits on the expression of ACC, p-ACC, AMPK, p-AMPK during 3T3-L1 preadipocyte differentiation. **(A)** Western blot analysis of p-AMPK, AMPK, p-ACC, ACC and β-actin expression. **(B)** The intensity of each band was determined using image **J.** The values represents means±SDs of triplicate experiments. Significant values are represented by asterisks (*** p < 0.001). Signification of the values is calculated in comparison to the values obtained using MDI-treated cells.

The MAPK and AMPK signaling pathways play a significant role in regulating PPARγ and C/EBPα expression during adipogenesis in 3T3-L1 preadipocytes. Therefore, the effect of the hexane fraction on these signaling pathways was investigated. To assess whether the inhibition of adipogenesis by the hexane fraction was associated with AMPK activation, the phosphorylation levels of AMPK and ACC were analyzed using western blotting (Fig 5A). The hexane fraction enhanced the phosphorylation of AMPK and ACC in a dose-dependent manner (Fig 5B). Specifically, treatment with the hexane fraction at a concentration of 125 µg/mL increased the phosphorylation of AMPK and ACC by 38% and 49%, respectively.

**Fig 6:**
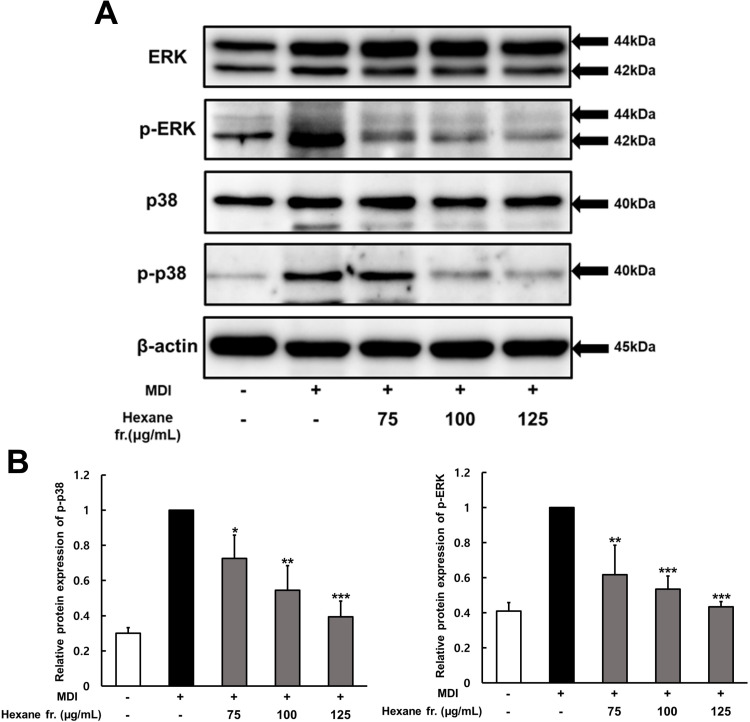
Effect of hexane fraction of immature Citrus unshiu fruits on the expression of p38, p-p38, ERK, p-ERK during 3T3-L1 preadipocyte differentiation. Two-day post confluent 3T3-L1 cells differentiated with MDI plus various concentration hexane fractions, respectively. **(A)** Western blot analysis measured the p38, p-p38, ERK, p-ERK and β-actin expression. **(B)** The intensity of each band was determined using image **J.** The values represents means±SDs of triplicate experiments. Significant values are represented by asterisks (*p < 0.05, **p < 0.01, *** p < 0.001). Signification of the values is calculated in comparison to the values obtained using MDI-treated cells.

The role of each MAPK pathway in hexane fraction-induced adipocyte differentiation was assessed through western blot analysis using specific antibodies against their phosphorylated forms. MDI treatment led to an increase in ERK and p38 phosphorylation relative to that in untreated cells (Fig 6), suggesting the activation of the MAPK pathway. However, treatment with the hexane fraction significantly reduced the MDI treatment-induced increase in both p38 and ERK phosphorylation. Specifically, the hexane fraction markedly suppressed ERK and p38 phosphorylation by approximately 56% and 61%, respectively, at 125 µg/mL compared with that in non-treated cells. These results suggest that the hexane fraction of ICE inhibited the MAPK signaling pathway by reducing ERK and p38 phosphorylation.

### Effects of ICE hexane fraction on lipolysis in 3T3-L1 adipocytes

**Fig 7:**
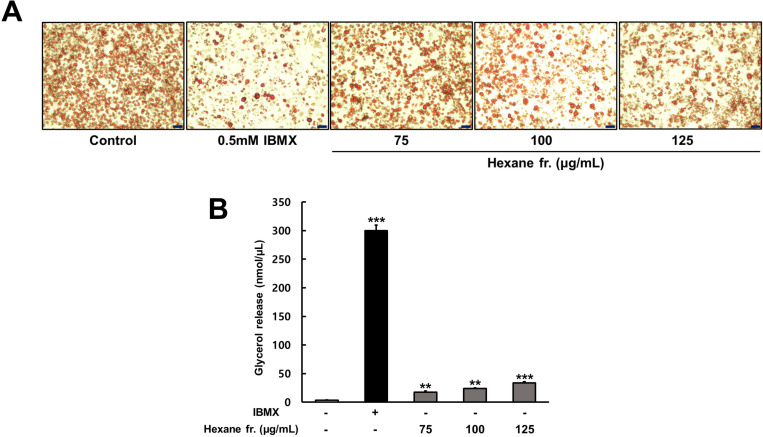
Lipolytic effects of hexane fractions in mature 3T3-L1 adipocytes. Differentiated 3T3-L1 cells were starved for 12 h and treated with 0.5 mM IBMX and hexane fractions (75, 100, 125 µ g/mL) for 48 **h. (A)** Differentiated adipocytes were stained with Oil Red O and stained triglycerides were presented at x200 magnification (scale bar: 100 μm). **(B)** Glycerol contents were determined in the culture medium. The values represents means±SDs of triplicate experiments. Significant values are represented by asterisks (** p < 0.01, *** p < 0.001). Signification of the values is calculated in comparison to the values obtained using non-treated cells.

To verify the dose-dependent effects of the hexane fraction on lipolytic activity, we measured the size and number of adipocytes, as well as the levels of free glycerol in the culture medium of 3T3-L1 cells treated with the extracts at concentrations of 75, 100, and 125 µg/mL. The size and number of adipocytes in the control group were large and dense; however, the positive control group treated with IBMX exhibited a significant reduction in both adipocyte size and number. In addition, the size and number of adipocytes in the experimental group treated with the hexane fraction decreased in a concentration-dependent manner (Fig 7A). Furthermore, the glycerol content in the IBMX-treated group was significantly higher than that in the control group. The glycerol levels in the medium of the hexane-treated group also increased in a concentration-dependent manner, with values of 13.6, 17.2, and 33.7 nmol/ µ L observed at hexane fraction concentrations of 75, 100, and 125 µg/mL, respectively (Fig 7B). These results suggest that the hexane fraction promoted lipolysis in differentiated adipocytes.

### Analysis of the active ingredients in the hexane fraction

**Fig 8:**
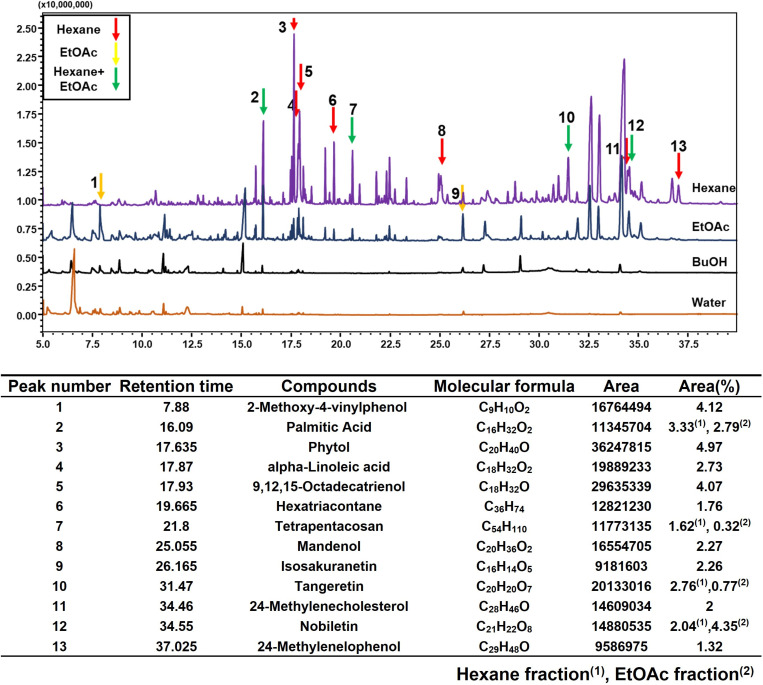
Gas chromatography-mass spectrometry (GC-MS) chromatogram obtained using hexane fraction of immature *Citrus unshiu* fruits.

GC-MS analysis was performed to identify the active components responsible for the observed anti-obesity effects of ICE (Fig 8). Furthermore, hierarchical and k-means clustering analyses were performed to identify the components of the hexane and EtOAc fractions that exhibit anti-obesity activity (S2 Fig). By comparing the obtained peaks with those in the GC-MS database (Wiley version 9), 47 and 38 compounds were detected in the hexane and EtOAc fractions, respectively, with 15 compounds common to both fractions. Based on peak area, 13 compounds including phytol, hexatriacontane, tangeretin, and nobiletin were selected as major substances.

## Discussion

Obesity is characterized by excessive lipid accumulation in adipose tissue, resulting from an imbalance between food intake and consumption. This condition manifests through hypertrophy, which involves an increase in adipocyte size, and hyperplasia, which refers to an increase in adipocyte number. Therefore, several mechanisms have been proposed to address obesity, such as reducing food intake, increasing energy expenditure, decreasing preadipocyte differentiation, lowering lipogenesis, and enhancing lipolysis [22,23].

Citrus extracts exhibit various biological and pharmaceutical activities [15,24]. Recent research has demonstrated that citrus polyphenols can revolutionize obesity management by inhibiting adipocyte differentiation and significantly reducing lipid accumulation within cells [25]. Moreover, previous studies have established that citrus extracts, such as *Citrus kiyomi x ponkan*, *Citrus limon*, and *Citrus sunki* can reduce the proliferation of 3T3-L1 cells and inhibit lipid accumulation [21,26,27]. However, despite the recognized presence of flavonoid compounds associated with anti-obesity effects in immature citrus, research on the anti-obesity effects of immature citrus has been limited. Therefore, we investigated the anti-obesity effect of immature citrus extracts on 3T3-L1 cells to explore their potential as complex functional food materials.

This study revealed that ICE and its hexane fraction suppressed the differentiation of preadipocytes into adipocytes and reduced lipid accumulation in 3T3-L1 cells (Figs 2 and 3). The inhibitory effect of ICE and its hexane fraction on lipid accumulation appears to be closely related to various bioactive compounds present in ICE, particularly polyphenolic compounds. These findings are consistent with numerous studies reporting that polyphenols and other bioactive compounds found in various natural products, including medicinal plants and fruits, can reduce lipid accumulation in 3T3-L1 cells [23,7].

During preadipocyte differentiation into mature adipocytes, transcription factors such as PPARγ, C/EBPα, and SREBP1 play key roles in promoting adipogenesis, leading to the expression of adipogenesis-related genes such as FAS and FABP4 (aP2) [28]. Therefore, we investigated the effects of immature citrus extracts on the expression of key adipogenic transcription factors and adipogenesis-related proteins. The hexane fraction of ICE significantly downregulated the expression of SREBP1, PPARγ, C/EBPα, FAS, and FABP4 in 3T3-L1 cells (Fig 4). These results are consistent with those of previous studies, which demonstrated that *citrus kiyomi x ponkan* and lemon extracts inhibited PPARγ, C/EBPα, and FAS expression in a dose-dependent manner in 3T3-L1 cells and high-fat diet-induced obese mice [27,7]. Therefore, our results suggest that ICE may exert its anti-obesity effects by suppressing the expression of genes involved in adipogenesis. Specifically, ICE may suppress adipogenesis by inhibiting the expression of PPAR and C/EBP, which are key transcription factors that promote adipogenic differentiation.

AMPK, a key regulator of energy balance, plays a significant role in obesity by inhibiting the differentiation of adipose precursor cells when activated [29]. Activated AMPK inhibits lipid synthesis by phosphorylating ACC, which suppresses FAS activity and subsequently reduces adipogenesis. Previous studies have also reported that many natural compounds inhibit mast cell differentiation by inhibiting the expression of FAS and adipogenic transcription factors [30]. Therefore, to determine whether ICE inhibits adipogenesis through the AMPK signaling pathway, we examined changes in the phosphorylation and expression of AMPK and ACC. ICE treatment led to a dose-dependent increase in the phosphorylation of AMPK and ACC, suggesting that AMPK activation is involved in the inhibitory effect of ICE on adipogenesis. These findings align with previous research indicating that *Citrus sphaerocarpa* and bitter orange extracts mitigate obesity by promoting AMPK phosphorylation in 3T3-L1 adipocytes and high-fat diet-induced obese mice [31,32].

Moreover, during adipocyte differentiation, various transcription factors such as PPAR and C/EBP are involved in the MAPK pathway, with PPARγ and C/EBP proteins being phosphorylated by ERK and p38 members of the MAPK signaling pathway [33,34].

Therefore, the effect of the hexane fraction of ICE on the activation status of ERK and p38 MAPK was assessed. ERK and p38 exhibited increased phosphorylation compared with those in control cells, indicating that the MAPK signaling pathway was activated (Fig 6). However, ICE effectively inhibited the MAPK signaling pathway by reducing the phosphorylation of ERK and p38. Therefore, ICE may be involved in inhibiting adipocyte differentiation by regulating the MAPK and AMPK signaling pathways.

Lipolysis is crucial for regulating the levels of TGs stored in fat deposits. This process occurs via activation pathways such as cAMP-dependent protein kinase A (PKA), hormone-sensitive lipase (HSL), and perilipin pathways, which are regulated by intracellular cAMP levels [35, 36].

Therefore, the effect of ICE on lipolysis in mature 3T3-L1 adipocytes was evaluated. ICE treatment led to a dose-dependent increase in glycerol content compared with that in control adipocytes. These results align with those of previous reports on sinensetin, nobiletin, and tangeretin, which demonstrated that these compounds promoted lipolysis via the PKA pathway in 3T3-L1 adipocytes [14,21,37]. These results suggest that the phenolic compounds present in ICE promoted lipolysis in mature adipocytes via the cAMP-dependent PKA/HSL pathway (Fig 7).

GC-MS analysis of the hexane fraction of ICE, which exhibited the strongest anti-obesity effect, identified phytol, hexatriacontane, 9,12,15-octadecatrienol, and nobiletin as the main components (Fig 8). Previous studies have demonstrated that hexane and n-BuOH solvent extracts from plants with anti-adipogenic activity mainly contain fatty acids, and their derivatives can inhibit adipocyte differentiation, often in combination with other phenolic substances [38,39,40]. Furthermore, plants from the Artemisia genus have been reported to produce flavonoids and phenolic compounds with anti-obesity effects [41]. Phytol and its metabolites have also been associated with anti-obesity activities [6]. Additionally, Alpha-linolenic acid has been shown to regulate lipid metabolism and related metabolic disorders in both adipocytes and animal models [42,43]. Moreover, nobiletin has been reported to inhibit lipid accumulation and attenuate obesity-induced inflammation through the NOB–ROR and IκBα/NF-κB pathways [44].

Based on these findings, phytol, fatty acids, and phenolic compounds in the hexane fraction of ICE are suggested as key contributors to its anti-obesity effects. Further studies are needed to confirm their roles and elucidate the precise mechanisms underlying these effects. In addition, considering this study and previous research, immature *Citrus unshiu* fruits show potential as an anti-obesity agent; however, their real-world applicability remains unclear. Therefore, additional animal and clinical studies are necessary to evaluate their bioavailability, safety, and optimal dosage.

**Fig 9:**
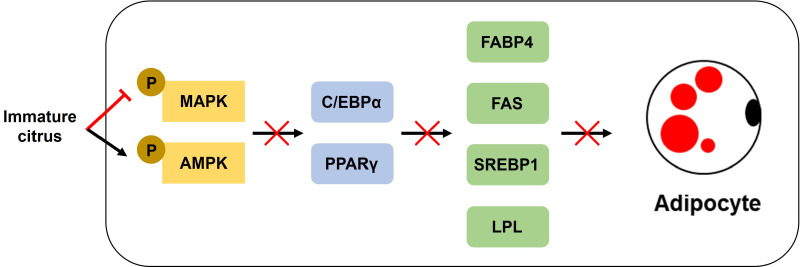
Suggested molecular mechanism for anti-adipogenic effect of ICE in 3T3-L1 adipocytes.

In conclusion, our results revealed that ICE and its hexane fraction exerted anti-obesity effects by inhibiting lipid accumulation. Notably, these effects were mediated by the suppression of the expression of adipogenesis-related genes and regulation of the AMPK and MAPK pathways in 3T3-L1 adipocytes (Fig 9). In addition, our results revealed that the phytol, fatty acids, and phenolic compounds (such as tangeretin and nobiletin) contained in the immature *C. unshiu* fruits were likely involved in the suppression of obesity. In light of these findings, further research will be conducted to develop anti-obesity functional foods derived from immature C. unshiu fruits.

## Supporting information

S1 Fig Effect of hexane fraction of immature Citrus unshiu fruits on triglyceride content in 3T3-L1 adipocytes. Intracellular TG content was quantified using a triglyceride quantitation assay kit on day 8 of differentiation.
(TIF)

S2 Fig Cluster analysis of compounds in each solvent fraction derived from immature citrus extract. (TIF)
