# Supplementary figures and images for: Immature Citrus unshiu fruit extracts inhibit adipogenesis in 3T3-L1 adipocytes via AMPK and MAPK signaling pathways
Source: PLoS One. 2025 May 8;20(5):e0322619. doi: 10.1371/journal.pone.0322619 (PMC12061173; doi:10.1371/journal.pone.0322619)

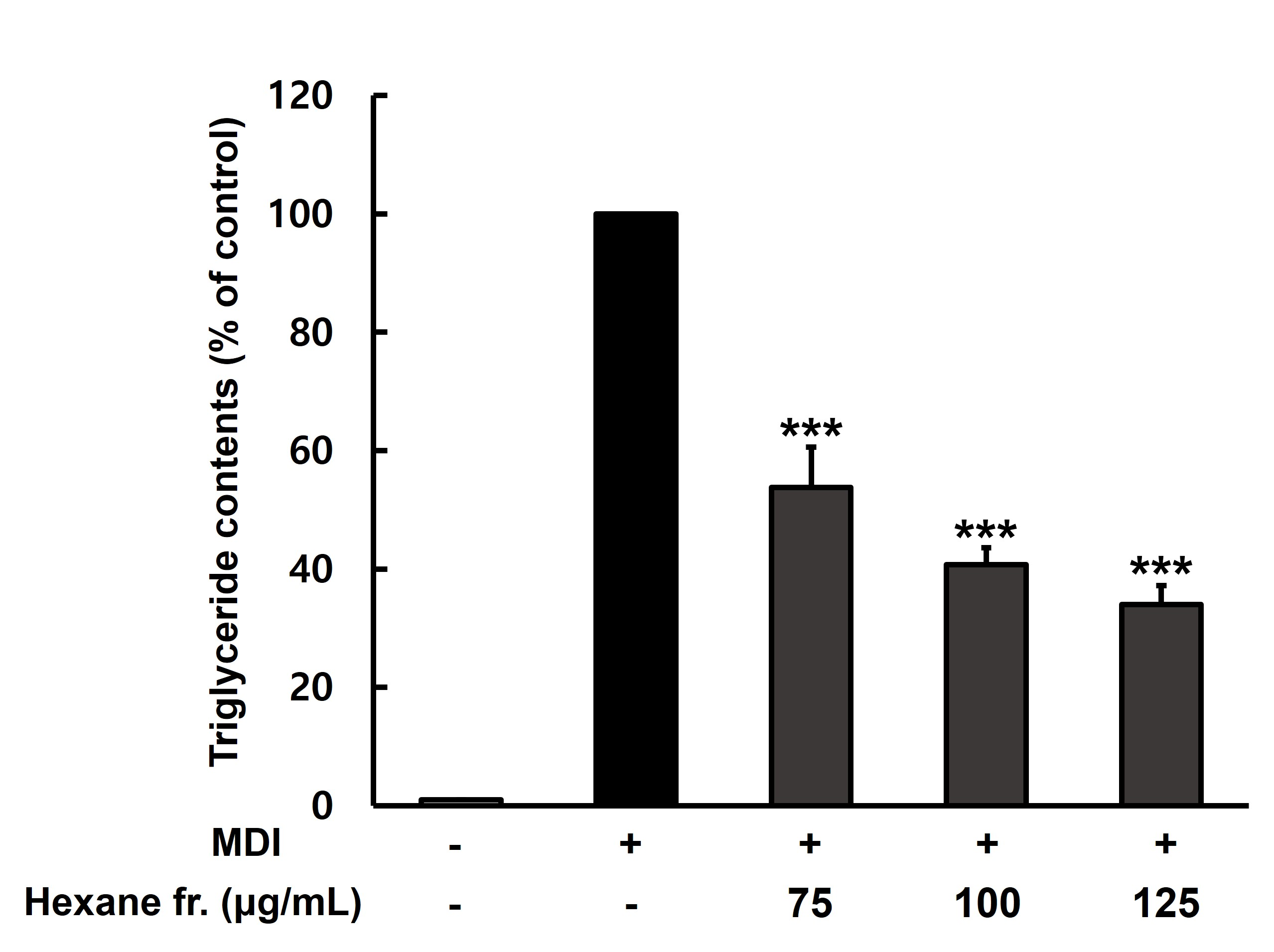

Supplement: S1 Fig — Intracellular TG content was quantified using a triglyceride quantitation assay kit on day 8 of differentiation. (TIF) [file pone.0322619.s001.tif]

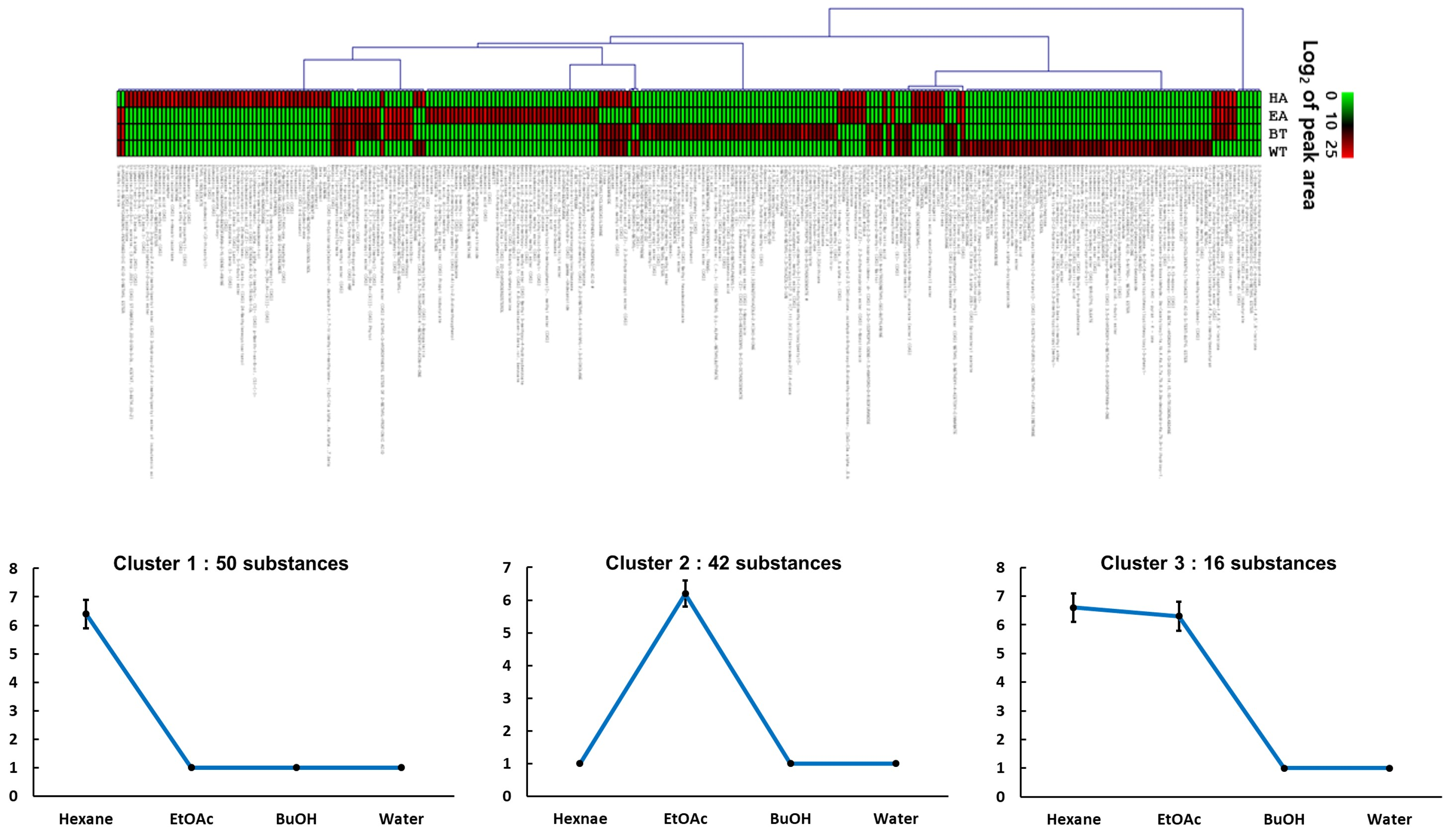

Supplement: S2 Fig — (TIF) [file pone.0322619.s002.tif]
